# Untimely Myocardial Infarction or COVID-19 Vaccine Side Effect

**DOI:** 10.7759/cureus.13651

**Published:** 2021-03-02

**Authors:** Zachary Boivin, Jennifer Martin

**Affiliations:** 1 Emergency Medicine, University of Connecticut Emergency Medicine Residency, Farmington, USA; 2 Emergency Medicine, Saint Francis Hospital, Hartford, USA

**Keywords:** covid-19, ultrasound (u/s), covid-19 vaccine, st-elevation myocardial infarction (stemi), point-of-care ultrasound

## Abstract

We present the case of a 96-year-old female, with no known cardiac history, who suffered a myocardial infarction (MI) one hour after her first Moderna coronavirus disease 2019 (COVID-19) vaccination. The patient was medically managed and discharged three days later. We are unable to attribute the cause of the patient’s MI to the Moderna vaccine unless further data are published. As healthcare providers, we need to be aware of attempts to correlate bad outcomes with the vaccine without substantiated data, and anticipate patient questions that may arise from these reports. Any research on the topic should be written carefully and avoid overstating the findings. If more reports of serious side effects in older adults are published, providers should consider additional screenings prior to COVID-19 vaccination.

## Introduction

The Moderna coronavirus disease 2019 (COVID-19) vaccine was granted emergency authorization use on December 18, 2020, based on strong phase three study data [1]. Moderna reported a 1% serious adverse event rate in both the vaccinated group and the control group [2]. These serious adverse events were defined as death, a life-threatening adverse event, inpatient hospitalization, a persistent or significant disruption to conduct normal life functions, or a congenital abnormality or birth defect [1]. They also reported no difference in thrombotic events between groups [2]. There are minimal published reports in the literature of serious adverse reactions to date [3,4]. This case report presents a potential serious adverse reaction and explores whether we can attribute a serious adverse reaction to the COVID-19 vaccine.

## Case presentation

An otherwise healthy 96-year-old female with a medical history of poorly controlled hypertension and a DNR/DNI code status was brought into the emergency department (ED) by emergency medical services (EMS) with chest discomfort that started approximately one hour after receiving her first dose of the Moderna COVID-19 vaccine. She had no other symptoms, specifically no shortness of breath, diaphoresis, or nausea. EMS transmitted an electrocardiogram (EKG) concerning for ST segment elevation in the inferior leads with reciprocal ST segment depression in the lateral leads. She had no history of cardiac disease or heart failure, reported no family history of heart disease, had never seen a cardiologist, and her medications consisted of hydrochlorothiazide (25 mg) daily for hypertension and a daily low-dose aspirin. She also was a non-smoker and had no surgical history. Family members confirmed her history and noted she had a history of medication noncompliance. Chart review showed multiple medical visits dating back approximately four years showing systolic blood pressures greater than 160 mmHg and normal diastolic blood pressures.

**Figure 1:**
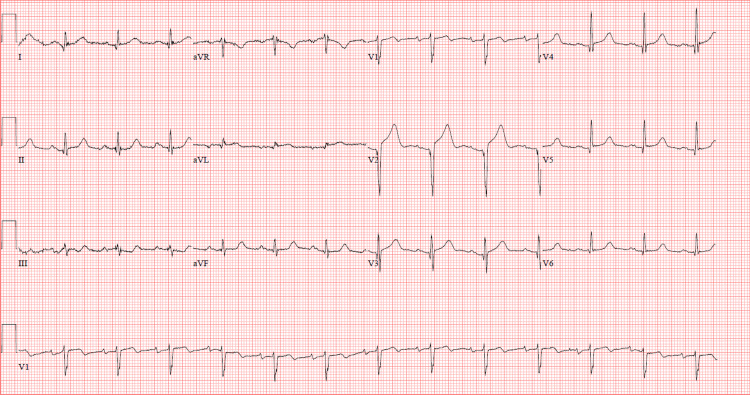
Initial emergency department EKG. EKG, electrocardiogram

Repeat EKG in the ED (Figure 1) showed ST segment elevation in the anterior leads of V2, V3, and avL, with no reciprocal ST segment depression. A point-of-care ultrasound performed by ED providers showed an anterior wall motion abnormality (Figure 2). The patient declined cardiac catheterization, and after consultation with cardiology, she was admitted to the hospital, started on a heparin drip, and a formal EKG was ordered. Her initial troponin was 0.07 ng/mL, with a peak of 2.63 ng/mL.

**Video 1:** Emergency department point-of-care ultrasound.

The formal EKG performed in the ED showed an ejection fraction of 35%, and an anterior and apical wall motion abnormality consistent with an anterior myocardial infarction (MI).

The patient’s hospital course was otherwise uneventful, and she was discharged home three days later.

## Discussion

This case explores an unfortunate MI after the Moderna vaccine. According to data from the Centers for Disease Control (CDC), over 800,000 people have MIs per year [5], and 33% or more occur in adults 75 years or older [6]. With older adults getting priority for the vaccine, we may continue to see older adults present with MIs to the emergency department after their COVID vaccination. This does not mean the COVID-19 vaccine is directly causing MIs, but perhaps it could be a contributing factor by placing increased demand on the heart.

The initial reports from the Moderna trial, which involved 30,351 participants 18 years of age or older, showed 92% of patients reported pain at the injection site, 70% reported fatigue, while 23% reported nausea and vomiting, 15.5% reported a fever, 19.8% reported axillary swelling or tenderness, and 23% reported nausea and vomiting. In the trial, 24.8% (7,520) participants were 65 years of age or older [2]. While Moderna considered these side effects minor, they could be significant stresses for older adults, especially those with preexisting comorbidities. In Norway, there was concern that the Pfizer vaccine was associated with increased mortality in older adults with 23 deaths, and investigation into these deaths suggested some of the listed side effects above might have caused the death of “frail patients” [7]. According to the CDC, there have been greater than 1,100 reports of death after the COVID-19 vaccine, with no indication of a link to the vaccine [8]. However, there is no published research on any of these cases, limiting our ability to understand the circumstances surrounding these deaths.

It stands to reason that older adults who have multiple comorbidities could be overwhelmed by side effects. In the case we presented here, the stress of getting the COVID-19 vaccination could have led to a demand ischemia in an older adult who had an unknown coronary artery plaque burden but a history of poorly controlled hypertension, leading to a cardiovascular event. These older adults, however, are also likely to perform poorly if infected with COVID-19 given their response to the vaccination. Our patient came to the ED one hour after receiving the vaccine, likely too early to experience any of the aforementioned side effects. Unfortunately, we were unable to confirm her coronary artery plaque burden as she declined a cardiac catheterization.

However, because many older adults have MIs, statistically some of those will happen soon after receiving the COVID-19 vaccine. This does not mean the COVID-19 vaccine caused the MI, but instead it could be coincidental occurrence.

## Conclusions

We report a case of an MI after the first dose of the Moderna COVID-19 vaccine. Until there are additional data released from the vaccine manufacturers, or there are an increasing number of case reports or case series detailing more serious side effects, we will be unlikely to determine whether there is a significant risk posed to older adults receiving the vaccine. In the meantime, healthcare providers should be aware that there might be false or overstated reports of danger from the COVID-19 vaccine, which could incite a rush to judgement by the general public. Any research on the topic should be written carefully and avoid overstating the findings. Additionally, as a precautionary measure, providers should consider additional screenings for older adults prior to COVID-19 vaccine administration if there continue to be reports of serious adverse events in older adults.
